# Lectures

**Published:** 1844-09-21

**Authors:** Benjamin Collins Brodie

**Affiliations:** Consulting-Surgeon of the Hospital


### CLINICAL LECTURES AND REPORTS.

### LECTURES

*Delivered in the Theatre of St. George's Hospital, in the session* 1843–44,

BY SIR BENJAMIN COLLINS BRODIE,

Consulting-Surgeon of the Hospital.

*Scirrhus of the breast. Circumstances under which an operation ought or ought not to be performed. Results of the operation. Danger of the operation. Effusion in the pleuræ generally the cause of death. Identity of the different forms of cancer.*

Gentlemen—If a scirrhous tumour of the female breast be left to take its course, it gradually increases in extent; it contaminates the neighbouring textures; it finally ulcerates, and, in the great majority of cases, the patient's life is terminated in three or four years from the commencement of the disease. Not only is life terminated thus early, but death is preceded by a very painful state of the ulcer. The ulcer is disposed to bleed and to slough, and the patient's life is rendered miserable. There is not a much worse way of leaving the world than that of being destroyed by an ulcerated scirrhus of the

Looking at these facts alone, you would say there is no doubt that the proper thing to be done is to remove the disease by an operation. But there is another order of facts which must be taken into account. In the large proportion of cases in which the operation is performed, the patient is still not alive two or three years afterwards; and, in a great number of cases, instead of the operation stopping the disease, it actually seems to hasten its progress. But, besides this, the operation in itself is not in every case free from danger.

Now these different orders of facts have led different surgeons, accordingly as they have looked at one or the other, to arrive at opposite conclusions as to the propriety of an operation. I have known some very excellent surgeons, among whom were the late Mr. Cline and Sir Everard Home, both men of great experience, who would scarcely ever consent to the operation for the removal of a scirrhous tumour of the breast, under any circumstances whatever. But then, I have known other surgeons, also very experienced men, who were in favour of the operation, perhaps in the majority of cases. And not only has there been this variety of opinion between different individuals, but I have found the opinion of the same individual to differ at different periods of his life. A very distinguished surgeon once said to me that he thought he would never perform this operation again, and yet that very surgeon, three or four years afterwards, strongly recommended the operation in a case in which I thought it would fail. This discordance of opinion only shows the difficulty with which the subject is beset, and if this difficulty has stood in the way of men of great experience in their profession, it may well stand in your way, who are only beginning your career. It appears to me, therefore, that it may be of advantage to you if I present some observations on the subject, and endeavour, as far as I can, to clear away the difficulty respecting the expediency and inexpediency of the operation.

This, then, constitutes the subject of the present lecture:—Under what circumstances is the operation for the removal of a scirrhous tumour of the breast proper, and under what circumstances is it improper.

It should, however, be observed, in the first instance, that while much depends upon the nature of the case, yet something depends upon yourself as to the mode of performing the operation. If there be a scirrhous tumour imbedded in the gland of the breast, and you remove the tumour, together with the part of the breast in which it is situated, leaving the remainder of the breast, according to my experience, the disease is certain to return; and this corresponds to a rule which I think applies to all cases of malignant disease—that is, that you have no security against the return of the disease unless you remove the whole of the organ in which it is seated. For instance, if there be fungus hæmatodes of the bone of the leg, the patient may have some chance if you amputate the thigh above the knee, but none if you cut through the tibia below the knee. If there be malignant disease of the femur, you have very little chance at all, unless you think it expedient to take out the thigh-bone at the hip-joint. I say, therefore, in cases of scirrhous tumour of the breast, if you perform the operation at all, where the tumour is imbedded in the breast, you must remove the whole of the organ. You may imagine that this is a thing very easy to be done, but you will not find it so in reality, for in amputating the breast, in a thin person, you will be very apt, if you are not extremely careful, to leave a small slice of the gland of the breast adhering to the skin, and I have no doubt that this small portion may, in some cases, form the nidus of future disease. The colour of the gland of the breast varies little from that of the surrounding adeps, the hæmorrhage causes confusion, and you must be careful in the dissection to keep the knife near the skin, not near the breast. But, in addition to this, in every case, when you have taken out the tumour, you should examine the surface, and see whether every part you have removed, is covered by healthy adeps. If it be not, look on the middle of the flap of the skin, and see whether any small portion of the breast has been allowed to remain there.

So far, then, the success of the operation may depend mainly on what you do; but now let us see what are the circumstances that are independent of any thing that you do, and which may induce you to think that there is no chance of the operation leading to an ultimate cure; and what are the circumstances that should lead you to hope that a permanent cure may be effected.

Scirrhous tumours of the breast may be divided into two classes; one, where there is a conversion of the gland of the breast itself into scirrhous structure, there being no well-defined margin; the other, where the scirrhous tumour is imbedded in healthy structure, as if it were altogether of a new growth, there being a distinct boundary to it.

In the first order of cases, not only does an operation never succeed in making a permanent cure, but it rather hastens the progress of the disease, and the patient generally dies in two or three years, if not before, of effusion of fluid into the cavity of the chest.

Where the skin is contaminated there is no chance of the operation making an ultimate and permanent cure; and it may be contaminated in various ways. Scirrhous tubercles form in the skin, here and there, at some distance round the tumour, while the intermediate portions of skin appear to be healthy, and then an operation will never lead to a cure; for you cannot remove all the contaminated skin. Where the skin is contaminated in this way, the progress of the disease is generally very rapid, and the patient dies in a short time from effusion within the chest. Sometimes the contamination of the skin developed itself in another manner. The skin becomes thickened and brawny, the pores are enlarged, as if you looked at them through a magnifying-glass, and you cannot pinch it up as you can healthy skin. This is also a very bad form of the disease. I have, however, performed an operation under these circumstances in two or three cases; the disease has always returned in the cicatrix directly, and the operation has appeared to hasten rather than to retard the fatal result. It does not matter to how small an extent the skin appears to be contaminated; if any portion of it is thus affected, the seeds of the disease are in the neighbourhood, and although your knife may divide the skin apparently healthy, yet it is not so in reality.

One effect of a scirrhous tumour of the breast, in a great number of cases, is to cause contraction of the lactiferous tubes which pass from different parts of the breast to the nipple, and this contraction gives rise to a drawing in or retraction of the nipple. I believe that this retraction of the nipple is to be regarded as very unfavorable to the ultimate success of an operation; for I suspect that the disease in these cases has always extended into the skin of the neighbourhood, and if you examine the skin in the neighbourhood of the nipple very carefully, you will generally find manifest indications of the disease in it.

In many cases of scirrhous of the breast, the skin is drawn over the tumour, and on looking at the patient, there is a sort of dimple over the tumour. Where this dimple is seen you may be almost sure that there is a scirrhous tumour beneath it, and when you examine it, you may feel it with the finger. The presence of this dimple is a very great objection to the operation, and there is little or no chance of a permanent cure. What is this indentation of the skin? I have dissected the parts, and I will tell you how it is produced. There is a small elongation of the disease which passes up from the scirrhous tumour, through the adeps, into the skin. There is a filament, as it were, of the disease, varying from a quarter to a half an inch in length, extending from the scirrhous tumour to the skin above it, and the presence of the dimple indicates that the disease is not confined to the breast, but that the skin is already contaminated.

As the disease advances, it contaminates the glands in the axilla. If the breast be inflamed, the glands in the axilla may be enlarged, just as glands may be enlarged from a boil or any other inflammation in the neighbourhood; but when there are large indurated glands in the breast, you may be sure ;that there is the same disease in the axilla—that the glands in the axilla are contaminated, and that there is no ultimate cure to be expected from an operation. You may say, remove the diseased glands from the axilla; I have done this myself, and I have seen it done by others, but I will tell you what always takes place. Perhaps there appears to be only one enlarged gland in the axilla, you attempt to remove it, but when you have got into the axilla you find that there are other glands contaminated in the same manner, though of too small a size to be perceived before.

I need hardly state that if the scirrhous tumour adheres to the parts below, to the pectoral muscle, or to the ribs, and the skin is ulcerated, there is no chance of a permanent cure from the operation.

You will sometimes find patients who, while they have a scirrhous tumour in the breast, have indications of some other form of malignant disease in other organs. One patient may have signs of malignant disease of the liver, another of the lungs, and another of the uterus. Of course, if there be any suspicion of the same mischief going on in internal organs, you will know that no permanent cure is to be expected by the removal of the diseased breast.

You must also take into account the state of the patient's health, her age, and her condition in other respects. If, for instance, an old woman labours under scirrhous of the breast, which is in a state of quiescence, you would never think of amputating the breast, because she may die first—the disease may out-last her.

Now, having taken away these cases, you will find, in practice, that there are very few left in which you will think it right to propose an operation.

What are the cases, then, in which an operation for the removal of the breast is proper? Where the skin is perfectly sound; where the nipple is not retracted; where there is no diseased gland in the axilla; where there is no sign of internal mischief; where there is no adhesion of the breast to the parts below; and where the patient is not very much advanced in life, I should say that there is a reasonable chance of an operation making a cure. I do not intend to say, that in all the excepted cases, there will be a permanent cure—far from it; but there will be in some instances, aud the chance of it may be sufficient to warrant yon in recommending the patient to submit to the operation. I have the satisfaction of knowing that several persons on whom I have operated under these circumstances, are now alive and well, but would certainly have been dead long since, had not I had recourse to it. As long since as 1832, I removed a breast affected with scirrhous tumour, and the lady was alive and well last year. Since the operation she has married and borne children. Last year I was called to see a lady on whom I performed the operation as long ago as 1830, and there she was, still alive and well.

But besides such cases as I have last described, there are others in which the operation for a scirrhous tumour connected with the breast may be proper. There is sometimes formed on the surface of the breast a hard tumour, which feels like scirrhus; on cutting into it, it looks like scirrhus, and I can give it no other name. It appears to be unconnected with the breast, but when you come to remove it you find that it is attached to the surface of the breast, just at one narrow corner. I have removed three tumours of this kind, leaving the breast uncut, except where I separted the tumours from it, and in each of these three cases the patient was alive and well some time afterwards. I do not know that in any one of these cases there was really a return of the disease.

Scirrhous tumours sometimes take place in the nipple, and I believe they are to be distinguished from similar tumours in the breast itself, and that there is a much greater chance of a permanent cure where they originate in the nipple than where they have their origin in the breast. A lady with a scirrhous tumour of the nipple, consulted several surgeons regarding it, and as the disease was quiet they all recommended that it should be let alone. After some time she came to London, and placed herself under the care of the late Mr. Rose, of this hospital. I saw her with him. The scirrhous tumour had been going on for some years, confined to the nipple, without coming to any harm, but it was now extending. We agreed that the operation should be performed, and Mr. Rose removed the breast. The breast appeared sound and the nipple alone diseased. The patient recovered, and was alive and well many years afterwards. A lady consulted me concerning what I must call a scirrhous tumour of the nipple, for it was hard, and presented the usual characteristics of scirrhous. It had ulcerated, but the breast seemed sound. She was a large, elderly woman, with a very large breast and a great deal of adeps. The removal of the breast would have been a frightful operation, and it is more than probable that her constitution would not have borne it. She was suffering great pain from the disease, and I applied chloride of zinc, and afterwards the caustic potassa, till I destroyed what appeared the whole of the disease of the nipple. This occurred three or four years ago, the wound healed, and the patient is alive and well at this moment.

The two last orders of cases are sufficiently distinguished from scirrhous tumours imbedded in the breast itself.

But here another question arises. Is there no reason for performing the operation for the removal of a scirrhous tumour of the breast than that of making a permanent cure? May it not be advisable to perform it sometimes in order to give the patient a respite, and to relieve her from present suffering? Undoubtedly it is, and I will mention some cases illustrative of this observation. A lady, about forty years of age, had a scirrhous tumour of the breast, and there was a cluster of diseased glands in the axilla; she came to me and the skin over the tumor appeared to be on the point of ulceration, so that the disease was proceeding to great mischief. I informed her that I was afraid the operation would not make a permanent cure, and that I could not recommend it. She inquired whether I had anything better to offer her, and I could not say that I had. She went away, but in two or three weeks came again, and said that she had consulted two or three other Surgeons, whom she named, and found that they were all of the same opinion, but that she had come to beg a favor, which was, that in spite of that, and to satisfy her, I would remove the breast. I asked what were her reasons, and she said that she was in these circumstances : that she had a daughter, an only child, eighteen years of age; and she knew she could not live long, but that it was a great object to her daughter that she should live to be her friend and adviser two years longer. It was for that reason and that only, that she wished to take the chance of the operation. There was no withstanding such an appeal as this, aud I removed the breast, but never thought of touching the glands in the axilla. There was no distinct return of the disease in the cicatrix, and the glands in the axilla did not much enlarge; but at the end of two years she was seized with symptoms of disease of the chest, effusion of fluid into the pleuræ, and she died. I may avail myself of this opportunity of mentioning that this is the most common way in which scirrhous tumours terminate life. Miliary tubercles, about the size of millet-seeds, form in the lungs, and then there is effusion of fluid into the pleuræ. A lady came to me with scirrhous tumour of the breast. Both the tumour and the breast were small, and I should have recommended the operation, but there were two or three hard glands in the axilla. I said to her, "You are not sufferiug much, I cannot recommend the operation; let the disease alone." A year afterwards she came to London again, and the tumour had now ulcerated, and the glands much increased. The ulcer in the tumour produced excessive suffering, and she was miserable. I did not remove it with a knife, but I used chloride of zinc and destroyed it. The sore healed, and some seven or eight months afterwards there was a tubercle formed in the cicatrix, which again ulcerated, and I destroyed that in the same way. She was thus enabled to go on with great comfort. After enduring the torture of scirrhous of the breast, she went on suffering nothing except at the time the chloride of zinc was applied, for a year and a half, but at the end of that time disease was established in the lungs, effusion took place into the pleuræ, and she died. A lady had a large malignant tumour in the breast: it was not exactly scirrhous, but approaching to it in its character. I did not think that an operation would lead to a permanent cure. By-and-by she came to me again, and now the tumour was ulcerated, and was very much enlarged. The skin had ulcerated, the ulcer was horribly painful, and her life was truly miserable. I said to her, "I am afraid you will not get an ultimate and permanent cure, but, suffering as you do, it is worth while to have your breast removed in order to relieve your present sufferings." It was a large tumour, which was one objection which I had to the operation. The operation was performed, and there was a good deal of bleeding. However, she recovered, and continued well upwards of three years. She had then some abdominal disease, and a tumour could be felt in the belly, which I concluded was of the same character as that in the breast. When I last heard of her she was supposed to be dying, and I presume that she is now dead ; but she was relieved from great suffering, and lived three years longer than she would have done had not the operation been performed. I may mention another case. A lady came to town with a large tumour in one breast. There was a fungus protruding, and in the centre of the fungus an opening, through which a probe could pass to the bottom of the tumour, and there was an enlarged gland in the axilla. Sir Astley Cooper saw her with me, and she was suffering a great deal from the ulcerated tumour. We agreed that she should have the breast removed, not that we expected a permanent cure, but to relieve her present sufferings. The breast was removed, the wound healed, and she had no return of the disease there, but a year afterwards her physician in the country wrote to say, that she had symptoms of some malignant disease going on in the chest, and she died of effusion into the pleuræ. There was another lady, with a small scirrhons tumour of the breast, which was very painful. She consulted me respecting it. I said that the operation would not make a permanent cure, but as she was suffering miserably she might as well have it removed. I removed it, and she was in comfort for many months.

There may, then, be cases in which your are justified in performing an operation for the removal of a scirrhous tumour of the breast, not with the expectation of effecting a permanent cure, but to obtain respite and relief to present sufferings. But here you must use some discrimination, for if the skin be thoroughly diseased I do not believe that in one case you will do any good ; the disease will return in the cicatrix so soon that the patient will derive no advantage whatever from the operation.

There is another circumstance to be taken into consideration when you are called upon to give an opinion as to the expediency or inexpediency of an operation. Is there any danger in the operation itself? It is commonly said that this is not a dangerous operation, but I can appeal to the experience of all surgeons who have had much to do with the operation, whether they have not had persons die from it—whether it is always free from danger. I know it is not. I have lost patients after the operation, and every surgeon, I know, has had the same misfortune. Here, I think, that something depends on the mode in which you perform the operation, and manage the patient both before and afterwards; and a great deal, also, depends upon circumstances not under your control.

The circumstances that are under your control are these. First, you should take care that there is as little hæmorrhage as possible at the time of the operation. Never give credit to those who stand up at an operation, and say that the patient has lost no more blood than will do him good. Hæmorrhage during an operation is a great evil, and is one of the chief causes of failure, not that the patient dies directly of hæmorrhage, but indirectly. It lays the foundation for erysipelatous and venous inflammation and other mischief some time afterwards. In addition to this, take care not to keep the patient very low before the operation. What we used to call preparing a patient for an operation, by low diet on all occasions, was very injurious. The patient need not be stuffed and crammed before an operation ; he should have his bowels emptied, but as to repeated purging and low diet, that is wrong both before and after any operation. An operation is a shock to the system, making a great demand upon the vital powers, and if you withhold the sustenance and stimulus to which the patient is accustomed, the constitution probably will not be able to bear the shock.

So far the success of the operation is to a certain extent under your control, but then there are circumstances not under your control that are unfavourable. For example, in a large fat woman, with an enormous breast, the operation is frightful; there is a large extent of surface, and there must be great hæmorrhage notwithstanding all your care. An old person will not sustain the operation like one less advanced in life. The operation is always attended with a certain degree of danger in a patient who, in other respects, is of delicate constitution. Those women whom you meet with in private practice, who have a small pulse, cold hands and feet, and are liable to attacks of hysteria, are always unfavorable for an operation, and especially one that is attended with a moderate loss of blood. In such women as these you must avoid an operation. But where the breast is small, where the patient is otherwise healthy, and not much advanced in life, and where you are careful not to starve the patient, either before or after the operation, and that there shall be as little blood lost as possible, there the danger of the operation is comparatively trifling.

I have thus spoken of the operation for the removal of scirhous tumour of the breast, but this organ is liable to other malignant diseases. The observations that I have made apply to the one case as well as the other, but I think that where malignant disease of the breast has the form of fungus hæmatodes the chance of ultimate success is even less than where it has assumed the form of scirrhus. Fungus hæmatodes is a worse form of malignant disease than scirrhus, and in the few cases which I have seen of it in the breast, where the tumour has been removed by operation, the patient has always died within a short time afterwards from some disease of the lungs and effusion into the pleuræ. But, after all, I believe that malignant disease is essentially of the same character, whether it assumes the form of scirrhus, or fungus hæmatodes, or pancreatic sarcoma. Whatever the name given to them by pathologists may be, I believe that malignant diseases are all nearly related one to another, and that the remarks I have made respecting one are applicable to the rest.

I will illustrate this last observation, which I think it is of importance in practice you should not forget, by mentioning some cases. A woman had a scirrhous tumour of the breast, attended with that brawny condition of the skin which I described as indicating a very bad form of the disease. There was a conversion of the glands into scirrhous structure, not a distinct tumour of the breast. She had also signs of disease of the liver and a discharge from the uterus. The woman died, and on examining the breast there was a well-marked scirrhous tumour; in the liver there was an equally well-marked tumour of fungus hæmatodes or medullary disease; and in the uterus that peculiar excrescence to which the late Dr. John Clarke gave the name of cauliflower excrescence of the uterus, and which he describes as a malignant disease. These three diseases, all of which are malignant, and to which different names have been given by pathologists, were associated in the same individual, and the preparations are now in the museum. But I have seen the same disease occur in succession, and I will mention a case in point. When I was a young man I went with Sir E. Home to perform a private operation. A lady from the country had a hard tumour, apparently in the abdominal muscles, which he removed, and when we came home and examined it we found that a portion of peritoneum adhered to it, and that it was a well-marked case of scirrhous tumour. The wound healed very well, but some time afterwards another tumour formed in the cicatrix, and began to enlarge. She came to London again, and put herself under Sir Everard Home. The tumour was now larger than the first he removed. He operated a second time, but this tumour had none of the characteristic structure of scirrhous. I can only describe it by saying it was like the fibrine of the blood, without colour; laminated something like the buffy surface of a coagulum of blood drawn during inflammation, and very slightly organised. The wound healed, but after a time another tumour formed in the cicatrix, and she again came to London. It was not thought worth while to remove this, it increased in size, occupied a great part of the belly, and she died. It devolved on me to examine the body, and the tumour now was entirely different in appearance from either of those which had been removed. It was a regular brain-like mass, a medullary tumour, or a tumour of fungus hæmatodes. In the one case three different kinds of tumour existed in the same individual at the same time; in the other three different kinds of tumour show themselves in succession. So you will sometimes remove a tumour from the breast in various parts of which you have a different structure.

There is a circumstance that ought to have been mentioned in an earlier part of the lecture, but which I accidentally omitted, and which is always to be taken into account whenever you have any doubt as to the expediency of performing an operation. It is very true that a scirrhous malignant tumour of the breast will, if left to itself, generally terminate the patient's life in three or four years, but very often it lasts much longer. I remember a lady of fashion who had a scirrhous tumour of the breast; she mingled in society, and nobody knew anything about it for several years; I believe ten or fifteen. I remember another lady who had a scirrhous tumour of the breast for twenty-five years, and she died at last, not from disease of the breast, but from effusion into the cavity of the chest. If you are in doubt about the expediency of an operation, and the disease be in an indolent state, the recollection of such cases as I have just mentioned should be sufficient to incline you to reject the operation. The chance of a patient living long with such a disease is not sufficient to make you throw away the chance of an operation where it is likely to be attended with advantage; but it is sufficient to make you decline an operation when other circumstances would lead you to doubt its propriety.—*London Lancet.*

